# Dengue–COVID-19 coinfection: the first reported case in the Philippines

**DOI:** 10.5365/wpsar.2020.11.3.016

**Published:** 2021-03-10

**Authors:** Angyap Saipen, Bernard Demot, Lowella De Leon

**Affiliations:** a Department of Internal Medicine, Baguio General Hospital and Medical Center.

## Abstract

The rainy season in the Philippines is from June to October; this is when the number of dengue cases typically increases. In 2020 during this time, the world was facing the threat of severe acute respiratory syndrome coronavirus 2 (SARS-CoV-2) infection. Coronavirus disease 2019 (COVID-19) and dengue viral infections have similar presentations and laboratory findings, including fever and thrombocytopenia, and there have been reports of coinfection with SARS-CoV-2 and arthropod-borne virus. Here, we report a case of SARS-CoV-2–dengue virus coinfection in the Philippines in a female aged 62 years, whose early symptom was fever and who was positive for SARS-CoV-2 and positive for dengue. Early recognition of such coinfection is important so that proper measures can be taken in the management of the patient.

Dengue fever is a mosquito-borne viral infection found mostly in tropical climates, including the Philippines. The clinical manifestations of dengue may include high-grade fever, headache, retro-orbital pain, muscle and joint pains, and rashes. In 2019, the Philippines had one of the highest numbers of reported dengue cases among countries in Asia and South-East Asia. (*1*) According to the World Health Organization (WHO), there were 55 160 cases of dengue in the Philippines from 1 January to 18 July 2020, a 66% reduction compared with the same period in 2019. (*2*) The endemic occurrence of dengue in 2020 coincided with the outbreak of COVID-19 infection. As of 11 August 2020, the Philippines has recorded 139 538 confirmed cases of COVID-19, making it the country with the highest number of cases in the WHO Western Pacific Region. (*3*) Here, we present the case of a female aged 62 years who presented at the emergency department with suspected COVID-19 and a suspicion of dengue fever; diagnostic tests were positive for both infections.

## Case identification

A female aged 62 years with hypertension who resided in the northern part of the Philippines presented at the emergency department on the evening of 4 August 2020 with body malaise and fever. Two days before her admission, the patient started to experience high-grade fever (highest recorded at 39.5 °C), with associated headache (frontoparietal in location, rated 5/10 and bandlike in character) and retro-orbital pain, generalized body ache, myalgia and arthralgia. There was no associated nausea, vomiting or blurring of vision. The patient had pain over the ankle joints, with no associated warmth or limitation of movement, and no rashes, cough or dyspnoea. The patient had self-medicated with paracetamol, which afforded temporary relief; however, her condition was persistent, with body malaise and weakness, prompting consultation at Baguio General Hospital and Medical Center Emergency Department.

The patient was admitted to the COVID-19 ward under the Internal Medicine Service, as a suspected case of COVID-19. The patient denied any history of travel outside the town or direct contact with anyone positive for COVID-19. She reported attending the public market three days before onset of her symptoms. At the emergency department, the initial physical examination of the patient was unremarkable except for decreased breath sound on the right basal lung field. Given the history of fever, the patient was managed as a suspected case of COVID-19.

**Figure 1:**
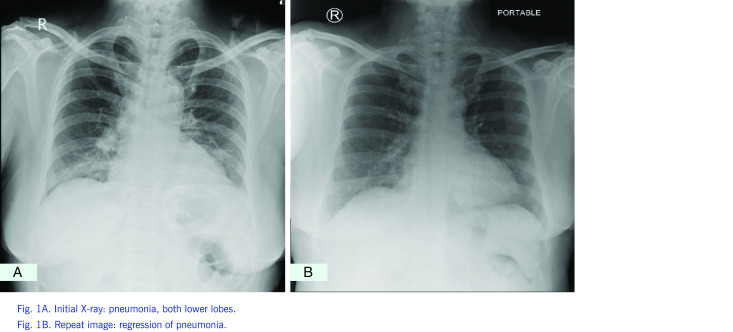
Chest radiographic image

Laboratory tests included reverse transcriptase polymerase chain reaction (RT–PCR) for COVID-19 (Sansure Biotech®), chest X-ray, complete blood count, blood culture, and inflammatory markers such as lactate dehydrogenase (LDH), erythrocyte sedimentation rate (ESR), ferritin, C-reactive protein (CRP), aspartate aminotransferase (AST) and alanine aminotransferase (ALT). The complete blood count initially revealed leukopenia at 3.16 × 109/L (neutrophils 75%, lymphocytes 18%), with haemoglobin and platelet counts being normal (140 g/L and 156 × 109/L, respectively). Chest X-ray revealed pneumonia on the right lower lobe of the lung (Fig. 1). There was no growth on blood culture. Markers of inflammation were elevated, including ferritin at 2156 ng/mL, ESR at 35 mm/hour, CRP at 18.73 mg/L and LDH at 317.92 U/L. Liver enzymes were elevated, with AST at 100.24 U/L ( × 2.86) and ALT at 65.11 U/L ( × 1.86).

[**insert** Figure 1]

## Course in the ward

In the COVID-19 ward, the patient’s social and environmental history was further investigated. A suspicion of dengue fever was considered after a comprehensive history had been taken from the patient, as she stated that dengue cases were present in her neighbourhood, with the latest case occurring one week before her symptoms commenced. The patient could not recall having any previous dengue infection. Rapid diagnostic tests (RDTs) for dengue non-structural protein 1 (NS1) antigen and Dengue Duo (WONDFO®) for immunoglobulins (IgM/IgG) were requested. The patient was positive for NS1 but negative for both IgM and IgG. The patient was then managed for suspected coinfection with dengue fever and COVID-19.

On the second day in hospital, the patient received a positive result from the COVID-19 RT–PCR (FAM/ORF1AB 36.46). A repeat of the complete blood count showed a sudden drop in the platelet count, from the initial 156 × 109/L to 85 × 109/L. There was persistent leukopenia at 2.85 × 109/L, with a notable increase in the lymphocyte count (from 18% to 37%). The patient consented to receiving favipiravir, started at 1800 mg (9 tablets twice a day) as a loading dose then reduced to four tablets twice a day for 13 days. Later tests showed further decreases in platelet counts, falling to 37 × 109/L on the fourth day of hospitalization (**Table 1**). A differential count of white blood cells (WBC) showed a further increase in lymphocytes to 49%.

**Table 1:** Summary of laboratory tests during the hospital stay (day 3 to day 11 of illness)

Laboratory tests		Day of illness^a^								
		Day 3	Day 4	Day 5	Day 6	Day 7	Day 8	Day 9	Day 10	Day 11
CBC	Hgb	140	-	147	152	136	140	137	-	-
	HCT	0.40	-	0.43	0.45	0.39	0.40	0.40	-	-
	WBC	3.16	-	2.85	6.43	9.41	9.62	8	-	-
	Neu	75	-	55	39	34	35	49	-	-
	Lym	18	-	37	43	49	48	42	-	-
	PLT	156	-	85	49	37	100	197	-	-
LDH		317.92	-	-	-	-	-	-	-	-
CRP		18.73	-	-	-	-	-	-	-	-
ESR		35	-	-	-	-	-	-	-	-
AST		100.24	-	-	-	-	-	-	-	-
ALT		65.11	-	-	-	-	-	-	-	-
Ferritin		2156	-	-	-	-	-	-	-	-
Blood A and B		(–)	-	-	-	-	-	-	-	-
COVID-19 RT–PCR		(+)	-	-	-	-	-	-	-	(–)
Dengue NS1		-	(+)	-	-	-	(–)	-	(–)	-
Dengue IgM/IgG		-	(–)	-	-	-	IgG (+)	-	IgG (+)	-

ALT: alanine aminotransferase; AST: aspartate aminotransferase; CBC: complete blood count; CRP: C-reactive protein; ESR: erythrocyte sedimentation rate; HCT: haematocrit; Hgb: haemoglobin; Ig: immunoglobulin; LDH: lactate dehydrogenase; Lym: lymphocytes; Neu: neutrophils; PLT: platelets; RT–PCR: reverse transcriptase polymerase chain reaction; WBC: white blood cells.

^a^ The patient developed clinical illness on 2 August 2020 and was admitted to hospital on 4 August 2020 (day 3 of illness).

**Figure 2:**
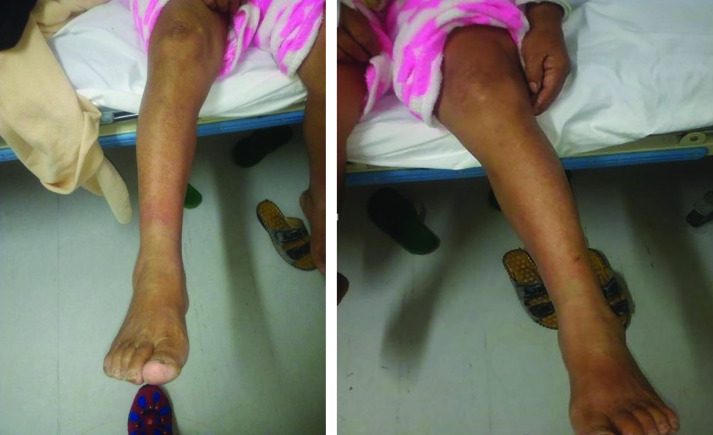
Chest radiographic image

A chest computed tomography (CT) scan showed posterior-basal pneumonia with features atypical of severe acute respiratory syndrome coronavirus 2 (SARS-CoV-2) pneumonia and minimal pleural effusion on the right. On the fifth day of hospitalization (day 7 of illness) maculopapular rashes appeared over the patient’s lower extremity, with some areas of erythematous petechial confluence and islands of normal skin (**Fig. 2**). A repeat test for dengue IgM/IgG on day 8 of illness was positive for IgG but negative for IgM. A repeat chest X-ray on day 9 of illness showed regression of previously noted densities in both lobes (**Fig. 1**). On the succeeding days, increasing trends in the number of platelets and leukocytes were noted. A further dengue IgM/IgG test on day 10 of illness yielded the same result as the previous test. After 10 days in hospital, the patient was discharged with her symptoms improved.

[**insert** Figure 2]

## Discussion

Fever is the most common symptom of COVID-19 infection. (*4*) In the case presented here, the patient had experienced two days of febrile episodes. Given her recent frequent travels to the public market, she was managed as a suspected case of COVID-19 and was isolated pending the result of a swab. However, the patient also manifested with typical symptoms of dengue, such as fever, generalized body ache, myalgia, arthralgia, retro-orbital pain and headache. (*5*) Because the patient could not remember any history of mosquito bite, we hypothesize that she was exposed to the dengue virus one week before her symptoms, at which time there were cases of dengue in her neighbourhood. The appearance of her symptoms coincided with the incubation period of the dengue virus – usually 4–10 days after the mosquito bite (*5*) – suggesting a high probability of dengue infection in this patient.

The decreasing numbers of WBCs and platelets also made the diagnosis of dengue likely. (*5*) However, thrombocytopenia and leukopenia are also common in COVID-19 patients. (*6*) The patient’s complete blood count result was consistent with both viral infections (i.e. leukopenia, with progressive thrombocytopenia occurring on succeeding days). The positive result from the dengue NS1 antigen test from the sample taken on the first day of hospitalization confirmed the dengue infection, although the IgG/IgM test at that time was negative.

Dengue RT–PCR, enzyme-linked immunoassay (ELISA) and viral culture are the ideal laboratory tests for the diagnosis of dengue. However, the only diagnostic tests available to the institution were the RDTs for dengue IgM/IgG and the NS1 antigen. The Handbook for clinical management of dengue notes that diagnosis of dengue infection is confirmed by the detection of the virus, the viral genome or NS1 antigen, or by seroconversion of IgM or IgG (i.e. from negative to positive). (*5*) Thus, the positive NS1 antigen test in this patient supported the diagnosis of dengue. The negative IgG and IgM may have been due to the timing of the collection of serum, given that dengue IgM serology has been shown to have low sensitivity during the early phase of dengue fever. (*7*) However, a negative antibody serology does not rule out dengue fever, especially when the dengue NS1 antigen test is positive. Samples taken from the patient on days 5 and 8 of hospitalization showed seroconversion of IgG but not of IgM. Given that the patient resided in a locality where dengue is endemic and cases have been reported, and had symptomatology and physical examination results suggestive of dengue fever, the combined positive NS1 test and the seroconversion of IgG improved the accuracy of the dengue fever diagnosis in this case.

In the natural course of dengue, IgM appears a few days following the onset of fever, followed by detectable IgG from day 5 onwards. The patient in this case study had a persistent negative IgM assay despite seroconversion of IgG, suggesting probable secondary dengue infection. Although the patient could not recall a previous dengue infection, she resides in a locality where dengue cases occur year-round; thus, it is possible that she had an undetected primary dengue infection. Primary dengue virus infections are often asymptomatic, and 90% of cases of dengue fever with symptoms occur following a second exposure. (*8*) Also, a low to negative IgM and a positive IgG for dengue may relate to recent secondary infection rather than being a marker of past infection. (*9*)

In the setting of a positive COVID-19 RT–PCR result, persistence of a positive IgM or IgG result on follow-up studies supports coinfection with dengue and COVID-19. (*10*) There have been few reported cases of such coinfections globally. Two cases reported were patients with a history of travel presenting with respiratory symptoms or rashes (or both). (*11*, *12*) However, there have also been false-positive results of dengue tests concurrent with SARS-CoV-2 infection. (*13*, *14*) In March 2020, two patients in Singapore were reported as having false-positive results from a dengue RDT but were later found to have confirmed SARS-CoV-2 infection. (*13*) Dengue IgM and IgG were noted to have cross-reactions with other flaviviruses such as malaria and leptospirosis. NS1 antigen testing is useful for differentiating between true dengue infections and false positives or coinfections, especially in resource-limited institutions, because the NS1 antigen is highly specific for dengue fever and has no cross-reactions, even with other flaviviruses. (*10*)

Studies on coinfections with SARS-CoV-2 and arboviruses are lacking, which is not surprising given that SARS-CoV-2 is a new disease. An extensive literature search on dengue and COVID-19 in the Philippines suggested that no previous case of dengue and COVID-19 coinfection has been reported in the country. The medical challenge of such coinfection lies in the similarity of the clinical and laboratory features of the two infections; (*13*) that is, fever, myalgia and headache, associated with leukopenia, thrombocytopenia and abnormal liver function. (*14*) Hence, it is important to consider the possibility of COVID-19 in patients positive for dengue and vice versa, since the result will affect management and prognosis. To avoid missing the diagnosis, we recommend testing for dengue infection once there is a high level of suspicion of dengue fever. At the same time, we recommend that testing for COVID-19 infection be considered in patients who present with history of fever or whose symptomatology is suggestive of infection by an agent other than SARS-CoV-2.

## ETHICS

Informed verbal and written consent was given by the patient.
